# Impact of an Invasive Insect and Plant Defense on a Native Forest Defoliator

**DOI:** 10.3390/insects7030045

**Published:** 2016-09-13

**Authors:** Claire M. Wilson, Justin F. Vendettuoli, David A. Orwig, Evan L. Preisser

**Affiliations:** 1 Department of Biological Sciences, University of Rhode Island, Kingston, RI 02881, USA; clairemariahwilson@gmail.com (C.M.W.); jvendettuoli@gmail.com (J.F.V.); 2 Harvard Forest, Harvard University, Petersham, MA 01366, USA; orwig@fas.harvard.edu

**Keywords:** plant-herbivore interactions, herbivore-herbivore interactions, plant defense, invasive species

## Abstract

Eastern hemlock (*Tsuga canadensis* [L.] Carriére) in the United States is threatened by the invasive hemlock woolly adelgid (*Adelges tsugae* Annand). The native hemlock looper (*Lambdina fiscellaria* Guenée) also appears to have played a role in previous population declines of this conifer. Although these two insects co-occur in much of the adelgid’s invaded range, their interactions remain unstudied. We assessed looper performance and preference on both uninfested and adelgid-infested foliage from adelgid-susceptible hemlocks, as well as on uninfested foliage from an eastern hemlock that is naturally adelgid-resistant. Larvae reared on uninfested foliage from adelgid-susceptible hemlocks experienced 60% mortality within the first two weeks of the experiment, and pupated at a lower weight than larvae fed adelgid-infested foliage. Despite differences in foliage source, this first look and strong pattern suggests that the hemlock looper performs better (pupates earlier, weighs more) on adelgid-infested foliage. In addition, trends suggested that larvae reared on foliage from the adelgid-resistant tree survived better, pupated earlier, and weighed more than in the other treatments. Larvae preferred adelgid-resistant over adelgid-susceptible foliage. Our results suggest that looper perform slightly better on adelgid-infested foliage and that plant resistance to xylem-feeding adelgid may increase susceptibility to foliar-feeding looper larvae.

## 1. Introduction

Trees and other long-lived plants must withstand multiple simultaneous or sequential attacks from an array of herbivores [1]. Many plants respond to these challenges through inducible defenses that can alter morphological structures, chemical composition, and volatiles [1,2,3]. Changes in plant tissues induced by an early-season attacker via plant defense or herbivore offense (sensu [4]) can alter host quantity or quality for subsequent attackers [5,6]. While some of these changes can deter attackers [7], others can increase the fitness of the later-arriving herbivore [8]. Because different herbivore feeding strategies induce different plant defense responses and plants are frequently attacked by more than one herbivore [9], it is important to conduct experiments that examine plant responses to multiple herbivore species [10,11,12].

Eastern hemlock (*Tsuga canadensis* [L.] Carriére) is a structurally-dominant and ecologically-important conifer found throughout the eastern United States [13]. As one of the most shade-tolerant conifers in the eastern U.S., *T. canadensis* has been called a ‘foundation species’ that provides critical habitat for a range of biota in both terrestrial and aquatic communities [13,14,15,16]. It is currently threatened by *Adelges tsugae* Annand (Hemiptera: Adelgidae), the hemlock woolly adelgid, which was introduced to Virginia in the 1950s and has caused widespread mortality of both eastern and Carolina hemlocks (*Tsuga caroliniana* [L.] Engelm). This invasive sessile hemipteran originated from Japan [17] and can kill trees in as little as two years, although some trees can survive over a decade after initial infestation [18,19]. The insect settles on the base of hemlock needles and feeds on xylem ray parenchyma cells [20] using a combination of at least four trophically-related enzymes [21]. This feeding leads to a cascade of physiological changes in the hemlock, including a systemic hypersensitive response [22], formation of false rings [23], and elevated salicylate levels [24,25].

The hemlock looper, *Lambdina fiscellaria* Guenée (Lepidoptera: Geometridae), is native to eastern North America and defoliates numerous tree species, including eastern and Carolina hemlocks. This lepidopteran has been linked to the mid-Holocene decline of hemlocks in the region [26,27]. From 1989 to 1993 over 200,000 hectares of Maine forests were defoliated [28], and in 2000, an outbreak in eastern Canada defoliated one million hectares of coniferous forest [29]. Early-instar larvae emerge in late May and feed primarily on new growth, where late-instar larvae consume old-growth needles [30]. Older larvae are wasteful feeders, consuming a small portion of one needle before moving onto another; at outbreak densities, this can cause rapid needle loss and kill mature trees within two years [31,32].

Although the two species co-occur in the southern portion of the looper’s native range and both insects can rapidly damage or kill their host at high densities, we are unaware of any research exploring their interaction. Because xylem-feeding *A. tsugae* nymphs settle and begin feeding before the needle-chewing *L. fiscellaria* larvae emerge [33], the adelgid may alter the quality of eastern hemlock foliage for later-arriving caterpillars. We assessed *L. fiscellaria* performance on adelgid-infested and uninfested foliage collected from adelgid-susceptible hemlock trees, and also on uninfested foliage gathered from a rare adelgid-resistant eastern hemlock. We also conducted bioassays to determine whether larvae prefer one type of eastern hemlock foliage over another.

## 2. Materials and Methods

We obtained hemlock looper eggs in spring 2014 from a rearing colony maintained by the Laurentian Forestry Centre of the Canadian Forest Service in Québec City, QC, Canada. The population was collected about 70 km north of Quebec City during an outbreak (summer of 2012). Movement of the eggs from Canada to the United States, and all of our subsequent research, was covered under APHIS (Animal and Plant Health Inspection Service) permit P526P-14-01875. The eggs were transported in coolers to the University of Rhode Island (‘URI’), where they were reared to pupation on three types of hemlock foliage: uninfested eastern hemlock (‘Susceptible’), adelgid-infested eastern hemlock (‘Susceptible + HWA’), and material from an individual eastern hemlock tree growing in a forested setting in Sussex County, New Jersey that previous research has found to be highly resistant to adelgid infestation (‘Resistant’; [34,35]). Although adelgid has been present in Sussex County for >20 years and nearby hemlock stands are filled with dead and/or dying trees, foliage from this tree consistently appears healthy and shows no signs of adelgid infestation [36].

Because of difficulties finding uninfested foliage on adelgid-susceptible hemlocks locally, we collected foliage from ten adelgid-susceptible trees (7–8 years old) purchased in spring 2013 from Vans Pines Nursery (West Olive, MI, USA; derived from seed collected in PA). These trees were not treated with pesticides at the nursery. These trees were potted from bare roots and grown outside (in pots containing field soil collected from below the trees used to provide Susceptible + HWA foliage) for a year at URI under insect mesh that protected them from adelgid infestation. Susceptible + HWA foliage was collected from ten trees (7–8 years old) growing on the southern edge of the URI campus that had been infested with adelgid for more than five years and that had not been fertilized or treated with pesticide in at least the past three years [37]. Previously, these trees were treated with soil drenches containing imidacloprid. Although we would have preferred to collect Resistant foliage from the clonally-propagated offspring of adelgid-resistant trees being grown at URI [34], we were concerned that removing foliage from these trees might compromise their usefulness in reforestation trials. Rather than sampling these propagated trees, we instead collected Resistant foliage from their parent tree (growing in Sussex County, NJ, USA). To ensure that looper larvae were encountering foliage of roughly similar quantity and quality, we only used pieces of foliage that were ~20 cm in length and contained both new- and old-growth needles. We collected new foliage when needed, mixed together with other foliage from the same treatment, and kept in a growth chamber (15 °C, 80% RH, 16L/8D cycle) until it was used to replace foliage. Larvae were never limited by amount of foliage available and new foliage was collected as needed.

We started the performance experiment on 12 June 2014 by placing two pieces of freshly-cut foliage from a given treatment into hydrated floral foam secured to the bottom of a 1L Ball Mason jar. Each piece of foliage was from a different main branch, randomly selected from all of the foliage collected for that treatment. Adelgids in the Susceptible + HWA foliage remained on the foliage. The top of each jar was covered with fine white mesh (~0.5 mm) that provided ventilation but prevented larvae from escaping. There were 16 jars per treatment, for a total of 48 jars; each jar was considered one replicate. As looper larvae hatched, they were randomly assigned to one of the 48 jars until each jar contained a total of five larvae (for an across-treatment total of 240 caterpillars). All 48 jars were held in the same growth chamber (15 °C, 80% RH, 16L/8D cycle). Foliage in each jar was replaced with fresh foliage as needed. Each piece of replacement foliage was randomly selected from the foliage collected for each treatment group. Jars were inspected daily, cleaned/watered as needed, and their position within the growth chamber was rotated weekly to control for possible microclimatic differences.

After all 48 jars were filled with five larvae each, any additional larvae that emerged were randomly assigned to one of three 5 cm × 25 cm × 15 cm plastic containers for use in the preference experiment (detailed below). The larvae density used in the experiment aligns with field observations of density [38]. Each container was assigned to one of three treatments (Susceptible, Susceptible + HWA, or Resistant), stocked with randomly-selected foliage from the appropriate treatment placed into hydrated floral foam, and covered with a white mesh top until preference tests were conducted. These containers were treated identically to the jars used in the performance experiment (i.e., same growth chamber, cleaning regimen, etc.).

### 2.1. Performance Experiment

We tested for differences in looper growth, development time, and survival on each of the three foliage types. Fifteen days after the experiment began, we removed the larvae from each jar and weighed them together using a Mettler Toledo scale (±0.001 mg). To determine survival, the number of living larvae was divided by five (the initial number of larvae in each jar) and mean weight by dividing the total weight by number of survivors. Larvae were then returned to their jar and growth chamber. These measurements were repeated at 33, 42, 57, and 74 days. After six weeks (42 days), each jar was checked every other day for pupae. Each fresh pupa was removed from the jar, weighed and the date recorded, and then frozen at −20 °C until they could be autoclaved per our APHIS permit. The experiment concluded on 12 October 2014, when the last larvae failed to pupate.

### 2.2. Preference Experiment

We tested whether second-instar looper larvae exhibited a preference for foliage of different types, and whether the foliage on which they were reared (‘natal foliage’) altered preference. All preference experiments were conducted using 9 cm petri plates that contained two 3 cm sprigs of fresh foliage from different treatments; for hydration, the stem of each sprig was inserted into a small hole in the top of a water-filled autoclave tube (~8 cm long). The two sprigs were on opposite sides of the plate and equidistant from a line drawn down the center on the plate underside. Each experimental replicate began with the two foliage types being randomly placed on the right or left of the petri plate. A single larva (reared on one of the two treatments) was then placed in the middle of the petri plate. The position of each larva (i.e., the foliage type to which it was nearest) was recorded after 0.5, 2, 4, 6, 9, and 24 h. After 24 h, each larva was removed and we ranked the amount of feeding damage (0% removed, 1%–25%, 26%–50%, 51%–75%, and 76%–100%) per needle for all needles on both sprigs. Gridded paper was used to guide the visual estimate of feeding damage.

We conducted two different preference experiments. In the first, we tested whether larvae (reared on either Susceptible or Susceptible + HWA) foliage exhibited a preference for Susceptible or Susceptible + HWA foliage. We recorded the percent time spent near each foliage type (over six observations) and total needle consumption over a 24-h window. We conducted an initial experiment on 9 July 2014 with six Susceptible-reared larvae and two Susceptible + HWA-reared larvae; we repeated the experiment one week later with seven Susceptible larvae and six Susceptible + HWA larvae, for a total of 13 Susceptible replicates and eight Susceptible + HWA replicates. Larval hatching times constrained replicate numbers. On 18 July 2014, we conducted a second experiment to determine if larvae reared on Susceptible or Resistant foliage exhibited a preference (both time and consumption metrics) for Susceptible or Resistant foliage. We tested 17 Susceptible-reared larvae and 19 Resistant-reared larvae.

### 2.3. Statistical Analysis

Jar (average weights/time to pupation of 5 larvae/jar) was used as the replicate for all data. Because our Resistant foliage (gathered from a single mature tree growing in a New Jersey forest) was not grown under the same conditions as our Susceptible and Susceptible + HWA foliage (each gathered from 7 to 8 years old trees grown on the URI campus), we present mean ± SE data from this treatment. We did not include Resistant foliage in the statistical analysis, and thus our statistical analyses are limited to comparing larvae reared on Susceptible and Susceptible + HWA foliage. We inspected all data for normality (Shapiro-Wilk test) and homoscedacity (Barlett’s test) prior to analysis; data were log-transformed when necessary to meet assumptions. Survival and larval weight over the first 74 days (=5 sampling periods) was analyzed using RM-ANOVA. Differences in time to and weight at pupation were analyzed using Welch’s *t*-tests. We analyzed data on percent pupation with a Kruskal-Wallis test, as the normality assumption was violated.

Two types of preference data were collected: (1) proportion of observations near each foliage type; and (2) needle consumption. Data on the number of observations (out of a total of six) a given larva spent near a foliage type per replicate was used to calculate the percentage of observations spent closer to each foliage type. We used a *t*-test to evaluate the hypothesis that caterpillars did not exhibit a preference (µ = 0.5), and to determine if the foliage type on which the larvae were reared (‘natal foliage’) affected their preference.

We also analyzed total needle consumption by calculating a feeding preference index (PI): PI_HWA_ = [(consumed area of Susceptible + HWA needles − consumed area of Susceptible needles)/total consumed area]; PI_RES_ = [(consumed area of Resistant needles − consumed area of Susceptible needles)/total consumed area] [39]. This preference index ranges from −1 (only consumed Susceptible foliage) to 1 (only consumed other foliage); zero values indicate no preference. We used *t-*tests to evaluate whether caterpillars exhibited a feeding preference. As mentioned, only data from the Susceptible and Susceptible + HWA treatments were statistically analyzed. Results from the Resistant foliage is presented as means ± SE. All analyses were performed using JMP 10.0 (SAS Institute, Cary, NC, USA).

## 3. Results

### 3.1. Performance Experiment

**Figure 1:**
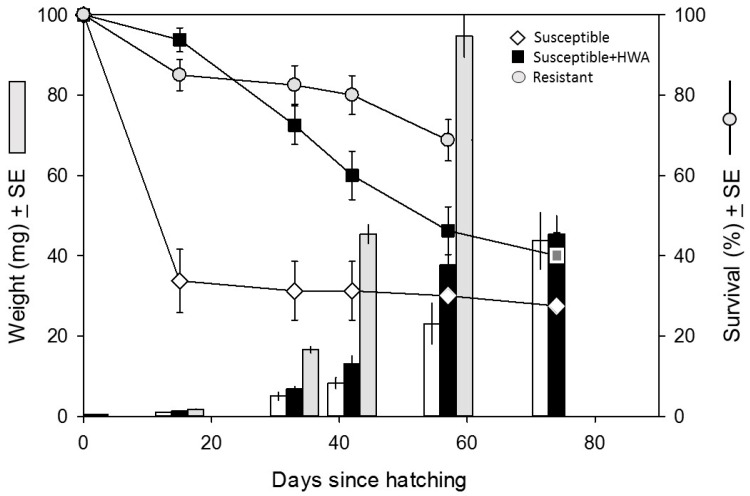
Mean ± SE weight (bars, left y-axis) and survival (circles, right y-axis) of larvae fed one of three different *Tsuga* foliage types. Data were collected in each treatment until pupation began. Susceptible = white diamonds/bars; Susceptible + HWA = black squares/bars; Resistant = gray circles/bars. N = 16 for each sampling point. Larvae on Resistant foliage pupated by day 57; larvae on Susceptible + HWA and Susceptible foliage by day 74.

**Figure 2:**
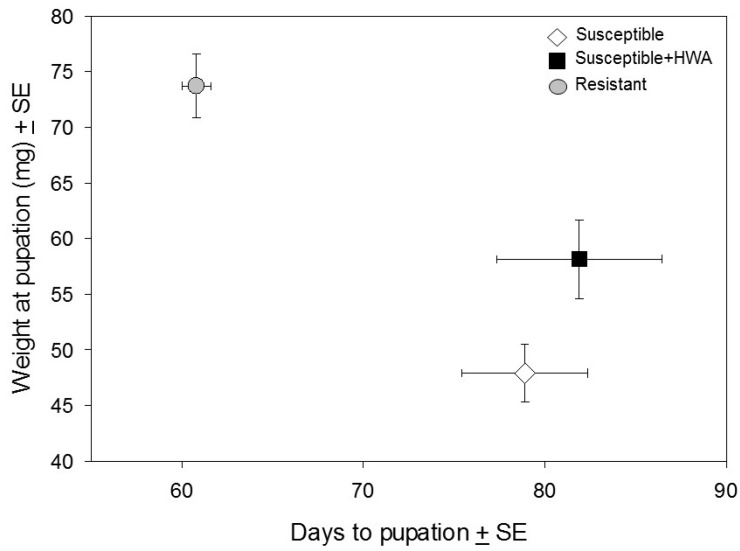
Relationship between days to pupation (x-axis) and pupal weight (y-axis) of *L. fiscellaria* larvae fed one of three different *Tsuga* foliage types. Susceptible = white diamonds; Susceptible + HWA = black squares; Resistant = gray circles. N = 16 for each sampling point.

Weight gain during the larval period did not differ between treatments (Figure 1; *F*_1,20_ = 0.8, *p* = 0.39), but larvae fed Susceptible + HWA foliage had a 20% higher pupal weight than those fed Susceptible foliage (Figure 2; *t*_18.5_ = 2.34, *p* = 0.030). There were also substantial between-treatment differences in survival across the five larval sampling periods (=77 total days; treatment: *F*_1,30_ = 14.8, *p* < 0.001). During the first 15 days of the experiment, 94% of larvae fed Susceptible + HWA foliage survived compared to only 34% of larvae fed Susceptible foliage (Figure 1). Despite their low initial mortality rates, larvae consuming Susceptible + HWA foliage experienced steady mortality throughout the rest of the experiment (time * treatment: *F*_4,27_ = 17.5, *p* < 0.001) and survival to pupation did not differ in the Susceptible + HWA (24% ± 2.4%) and Susceptible (20% ± 3.5%) treatments. Both treatments also had a similar time to pupation (*t*_18.7_ = 0.53, *p* = 0.60).

Larvae reared on Resistant foliage gained weight much more quickly than larvae in the other two treatments (Figure 1). The survival rate to pupation in the Resistant treatment was three times that of the other two treatments (66% ± 2.7% versus 22% ± 2.9%, respectively), and larvae in this treatment pupated >20% faster than larvae in the Susceptible treatment (61 ± 0.8 days versus 79 ± 3.5 days; Figure 2).

### 3.2. Preference Experiments

**Figure 3:**
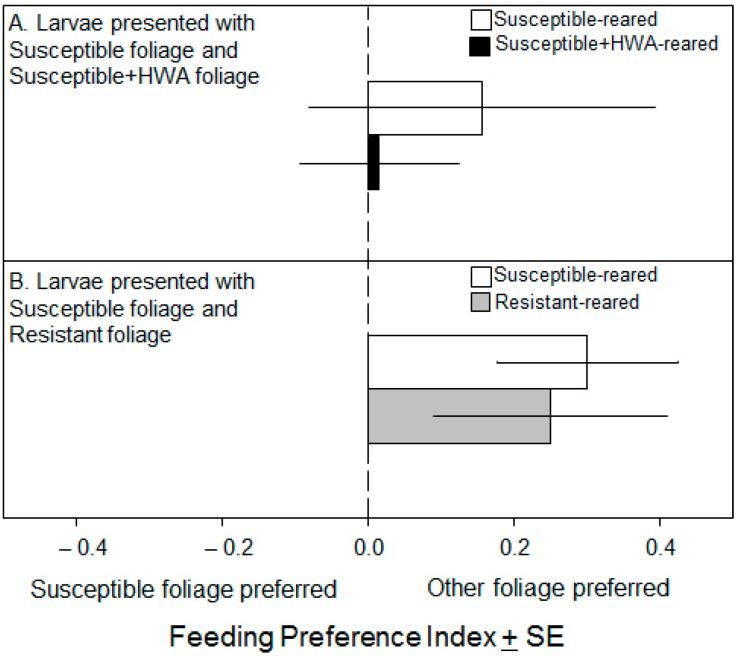
(**A**) Feeding preference index (−1: only ate Susceptible foliage; 0: no preference; 1: only ate other foliage type) of larvae reared on Susceptible (white bars; *n* = 7) or Susceptible + HWA (black bars; *n* = 6) natal foliage; and (**B**) Susceptible (white bars; *n* = 17) or Resistant (gray bars; *n* = 19) natal foliage.

When presented with a choice of Susceptible or Susceptible + HWA foliage, Susceptible-reared larvae were found near Susceptible + HWA foliage in 69% ± 8.0% of observations (*t*_12_ = −2.41, *p* = 0.03). When presented with the same choice, Susceptible + HWA-reared larvae were found near Susceptible + HWA foliage in 73% ± 8.8% of observations (*t*_7_ = −2.58, *p* = 0.04). Although both types of larvae spent significantly more time near Susceptible + HWA foliage, they consumed similar amounts of both foliage types and natal foliage did not affect needle consumption (*t*_8.68_ = 0.53, *p* = 0.61; Figure 3A).

When presented with a choice of Susceptible or Resistant foliage, both larval types spent a similar number of observations near both foliage types (Susceptible-reared larvae: 56% ± 8.9%; Resistant-reared larvae: 58% ± 8.4%); these means were not, however, statistically compared (see rationale in Materials and Methods). Both Susceptible- and Resistant-reared caterpillars had feeding preference indices >0, indicating that they consumed more Resistant foliage (0.30 ± 0.12 and 0.25 ± 0.16, respectively).

## 4. Discussion

We found that infestation by the hemlock woolly adelgid provided a moderate benefit to hemlock looper feeding on adelgid-susceptible eastern hemlock. Larvae reared on Susceptible + HWA foliage had much higher early-instar survival (Figure 1) and pupated at a larger weight than larvae reared on Susceptible foliage (Figure 2). Specifically, death rates within the first two weeks of the experiment were 66% for Susceptible-reared larvae versus 6% for Susceptible + HWA-reared larvae. In contrast to Susceptible-reared larvae, larval survivorship on Susceptible + HWA foliage declined at a relatively stable rate throughout the experiment (Figure 1). Although larval survival was always higher on Susceptible + HWA foliage than on Susceptible foliage, this difference was not significant at pupation; the fact that larvae fed Susceptible + HWA foliage pupated at a larger weight, however, suggests that this foliage was of a higher quality.

The differences in looper performance may result from adelgid-induced changes in plant defense induction. Feeding by first-instar adelgid crawlers substantially alters the tree’s resin profile [24,25]. A 10- to 100-fold increase in methyl salicylic acid, a compound involved in the systemic acquired resistance signaling pathway for sucking herbivores [40], was noted following *A. tsugae* feeding in adelgid-susceptible trees [24]. Although these fluctuations are more pronounced in the twigs where the adelgid feeds, they were also apparent in the needle volatile profiles. Because activation of the salicylic acid (‘SA’) pathway suppresses the induction of jasmonic-acid (‘JA’)-based defenses that are particularly effective against chewing herbivores, it can increase the vulnerability of SA-induced plants to this herbivore guild [10,41,42]. Early-instar larvae are more sensitive to host quality and defense than late-instar larvae [43], and the high early-instar mortality of loopers feeding on Susceptible foliage might reflect the rapid induction of JA-based plant defenses specific to chewing herbivores [44,45]. Our results may thus be consistent with previous work showing that feeding by phloem-feeding insects can benefit caterpillar development and survival [10,46].

A previous study that reared hemlock looper on eastern hemlock foliage started at the same time as ours (10 and 12 June, respectively) and with the same parental biotypes (reared on balsam fir, *Abies balsamea* (L.) Mill.) but found that the larvae matured more quickly and pupated at a larger weight (~89 mg [47]). Since the Hebert et al. [47] study and our work are apparently the only two reports of looper performance on hemlock, it is impossible to say which represents ‘normal’ values. It is worth noting, however, that the study also found substantial differences between island and mainland looper populations fed eastern hemlock, which pupated at ~73 mg and ~105 mg, respectively [47]. This suggests that developmental indices can vary substantially between looper populations, and may partially explain the differences between the two studies.

While adelgid infestation had a moderate effect on looper development, larvae fed Resistant foliage did much better than their counterparts in the other two treatments. Specifically, *L. fiscellaria* larvae fed Resistant foliage were more likely to pupate, mature quickly, and weigh more as pupae than larvae in the other two treatments (Figure 2). The preference bioassay also showed that larvae consumed more Resistant foliage than Susceptible foliage regardless of the foliage type on which they were reared (Figure 3B). Differences between the foliage in this treatment and the others (geographic location, single versus multiple trees, mature tree versus saplings) dissuaded us from statistically analyzing data from this treatment; there are, however, several reasons to believe that results from Resistant foliage are ecologically interesting. First, although foliar age (e.g., new- versus one-year growth) significantly affects foliar chemistry in conifers [48,49], a study addressing the impact of tree age (e.g., saplings versus mature trees) on *T. canadensis* foliar chemistry found only modest differences (i.e., no difference in constitutive levels of monoterpenoids sesquiterpenoids and benzenoids) between similarly-aged foliage [25]. Second, a related study compared the terpenoid profiles of adelgid-susceptible and -resistant *T. canadensis* (the latter group including the tree used in the current study) in the field and in a common-garden setting over an 18-month period [34]. The field trees were located in New Jersey, while the common-garden trees (saplings produced from rooted cuttings derived from the field trees) were grown for six years in RI at the URI greenhouse. This work found that terpenoid concentrations in the current-year-growth of NJ-grown mature trees were very similar to those of the RI-grown saplings, suggesting that in this case neither plant age nor geographic location substantially altered foliar chemistry.

While the potential for pseudoreplication (i.e., treating multiple pieces of foliage from a single source tree as statistically-independent replicates) still precludes the statistical analysis of Resistant data, it is nonetheless interesting to speculate why *L. fiscellaria* performance was so much better in this treatment. The study exploring terpenoid profiles of adelgid-susceptible and -resistant *T. canadensis* found that, although there was no difference in foliar chemistry, twigs from adelgid-resistant hemlocks had terpenoid concentrations twice that of twigs from susceptible hemlocks [34]. Resistant hemlock protected from adelgid infestation showed the same pattern as adelgid-exposed plants, suggesting that high twig-level terpenoid concentrations are constitutively expressed in adelgid-resistant eastern hemlocks [34]. Terpenoids are integral to herbivore defense in hemlocks and other conifers [50], and a range of studies have shown that plant defense against chewing herbivores can be altered by the presence of sap feeders, root feeders, and other herbivore guilds [5,51]. Future research in this area might address whether constitutive investment in twig-level defense interferes with the ability of adelgid-resistant hemlocks to induce anti-folivore defense.

The results of our work are interesting in light of the recent evolutionary history of hemlock in eastern North America. During the mid-Holocene, outbreaks of hemlock looper coincided with a dramatic decline in eastern and Carolina hemlock densities that restricted both species to a few isolated refugia [26,27]. Although the two species eventually re-colonized their historic range, continued looper outbreaks and the absence of native sap-feeding herbivores likely selected for effective anti-folivore defense [52]. This explanation is consistent with the fact that eastern and Carolina hemlock, although not closely related [53], share a similar terpenoid profile that differs substantially from those of five other *Tsuga* species [54], all of which are attacked by native sap-feeders [52]. Despite the genetic bottleneck imposed by the mid-Holocene decline of *T. canadensis*, the terpenoid profiles of its commercially-available cultivars exhibit considerable variation [55]; future research might explore the genetic basis of defense against folivores and sap-feeders in both adelgid-resistant and -susceptible *T. canadensis*.

While our results raise a number of interesting questions, we cannot completely exclude the possibility of pre-existing differences between the different types of hemlock foliage used in our experiment. The most obvious difference is between Resistant foliage (collected from a single mature tree growing in a New Jersey forest) and our Susceptible and Susceptible + HWA foliage (each collected from ten young trees growing on the campus of the University of Rhode Island). Our concerns about this comparison were sufficiently great that we chose not to statistically analyze the differences between these three treatments; even our Susceptible and Susceptible + HWA foliage, however, may also have had pre-existing differences. While the trees from which each set of foliage was collected were roughly the same age and grown without fertilizers or pesticides under apparently identical abiotic conditions, we cannot rule out the possibility that the two sets of plants differed from each other in factors other than adelgid infestation. We also lacked the resources necessary to sample foliar concentrations of jasmonic and salicylic acid, information necessary to determine whether adelgid infestation produced the hypothesized changes in plant defensive chemistry. Finally, some of our preference experiments had fairly low levels of replication (e.g., eight larvae) and would have benefitted from higher numbers. Each of these concerns is potentially important; given the absence of prior work addressing the interplay between these two economically- and ecologically-important herbivores, however, we nonetheless feel that our findings provide a valuable ‘first look’ at an intriguing system that should spur additional work.

## 5. Conclusions

The rapid adelgid-induced decline in eastern hemlock, a foundation species in eastern North American forests, underlines the importance of understanding how interactions between the adelgid and other herbivores impact hemlock. The fact that looper larvae performed slightly better on adelgid-infested foliage and much better on adelgid-resistant hemlocks suggests the possibility that adelgid-driven selection may initially increase this species’ vulnerability to looper outbreaks.
